# Italian validation of the situational Brief Cope Scale (I-Brief Cope)

**DOI:** 10.1371/journal.pone.0278486

**Published:** 2022-12-01

**Authors:** Ramona Bongelli, Alessandra Fermani, Carla Canestrari, Ilaria Riccioni, Morena Muzi, Alessia Bertolazzi, Roberto Burro

**Affiliations:** 1 Department of Political Science, Communication and International Relations, University of Macerata, Macerata, Italy; 2 Department of Education, Cultural Heritage and Tourism, University of Macerata, Macerata, Italy; 3 Department of Human Sciences, University of Verona, Verona, Italy; University of Foggia: Universita degli Studi di Foggia, ITALY

## Abstract

The Brief COPE (Coping Orientation to Problems Experienced) inventory is 14 faced scales used to assess coping strategies. It has been administered on different population samples and translated into several languages. Nonetheless, the Italian validation of its situational format is missing, and the present study aimed to fill this gap. To this end, the original English version of the scale was translated into Italian and administered to a sample of 682 Italian healthcare workers (HCWs), during the first wave of COVID-19. The Exploratory and Confirmatory Factor Analysis (EFA and CFA) were performed and led to the validation of the Italian Brief COPE (I-Brief COPE) scale, consisting of 21 items, loading properly on 6 factors, which range from activation (F1) to deactivation (F2), via social support (F3), humorous reframing (F4), religious/spiritual reliance (F5), substances use (F6). The six factors identified represent, according to our analyses, the relevant dimensions of coping in a stressful situation such as COVID-19. The results of this study reveal that the I-Brief Cope can be considered as a valid tool for measuring coping strategies in facing stressful, unpredictable, and damaging events.

## Introduction

Coping strategies can be defined as cognitive and/or behavioral efforts that subjects make to manage specific external and/or internal demands, which are evaluated as exceeding personal resources [1] or, more simply, as processes of response to stressors [2].

Coping has been the object of numerous studies since the 1960s (e.g., [1–4]), and much of this work has led to the identification and classification of coping strategies, primarily distinguished into dichotomous pairs (as many authors argue, including [5,6]), on the basis of their *focus* (the problem or the emotion) or their *mode* (approaching or avoiding) or the *outcome* they achieve (adaptive or maladaptive). Thus, in some of these researches, coping strategies have been presented as differentiated in *problem-focused*, i.e., aiming at actively responding to a stressful situation, by attempting to modify or eliminate the sources of stress, and *emotion-focused*, i.e., aiming to reduce, manage or regulate emotional responses related to the stressful situation—especially when the situation is considered as not modifiable—in the attempt to maintain an emotional equilibrium (e.g., [4,5,7–9]). In some others works, coping strategies have been instead distinguished in *approach* or *active*, i.e., aiming to directly face with the stressor and its related emotions, and in *avoidance strategies*, i.e., aiming to deny, minimize, or avoid dealing with stressors and confronting the problem [1,10–13]. In further studies, coping strategies have been classified as *adaptive*, i.e., characterized by more probability of obtaining a desirable result, and *maladaptive*, i.e., characterized by more probability of not obtaining a desirable result and, vice versa, by more probability of obtaining an undesirable result (e.g., [14]).

According to Carver et al. 1989 [11], this last distinction must consider the individual and the situation. Some coping responses may indeed be beneficial for some people in some situations, and not beneficial for other people or in other situations. In other terms, a certain coping strategy may not be maladaptive in itself, but it may become dysfunctional if it is used for a long time even when other strategies would be more useful. For example, seeking out emotional social support can be thought of as a *double-edge sword*: it is adaptive if it is able to reassure the subject and to foster a return to problem-focused coping; it could be maladaptive if used only for venting one’s feelings [11]. Although it is therefore impossible to distinguish in advance adaptive coping from maladaptive one, it is possible to affirm that adaptive coping is that mode of dealing with stressor for which the subject can adopt those strategies that best fit the level of controllability of the situation, maladaptive coping is the opposite [15].

Irrespective from these classifications, there is a widespread agreement that the use of specific coping strategies is related to multiple situational and personal variables, first and foremost by *the nature of the stressful event*, by the perceived levels of *controllability*–i.e., how much the stressful event is perceived by the subjects as controllable or uncontrollable (e.g., [1,11,16–18])–, and by *subjects’ personality traits* (e.g., [11,19–23]).

In the last two years, studies on coping have enormously increased because of the COVID-19 pandemic. Even though the virus spread was a cataclysmic stressor [24], it differently struck different areas of the world (i.e., it spread differently in terms of timing and intensity), and differently impacted on specific categories of individuals, such as *health care workers* (e.g., [25–29]); *elderly people* (e.g., [30–36]); *adolescents and young students* (e.g., [37–39]), *vulnerable patients* (e.g., [40–43]) etc., who had to cope with specific stressful aspects of the pandemic and/or of its related lockdowns: lack of medical protocols and sense of helplessness, fear of contagion, loneliness, isolation, fear of not being able to access health services—due to lockdowns—and not being able to get treatment for diseases other than COVID-19, etc. As in other stressful situations, perception of control and personality traits seem to have played an important role in the adoption of specific coping strategies. Specifically, as for healthcare workers, significant associations between higher levels of perceived control towards the pandemic and problem-focused coping styles have been found (e.g., [44]), as well as between specific personality traits and specific coping strategies (e.g., between neuroticism and dysfunctional coping strategies [45]).

### Coping measures

Although many instruments have been developed over time to measure how individuals cope with stressful situations, according to Kato [46] two are the most frequently used: the *COPE* (Coping Orientation to Problems Experienced) [11], and the W*ays of Coping Questionnaire* [47,48].

Unlike other situation or stress-specific coping scales (such as the Pain Coping Questionnaire [49], the Interpersonal Stress coping scale [50], the Coping self-efficacy scale [15], the Pandemic Coping Scales [51], or the Humor Coping Scale [52]—just to mention a few),—the COPE [11] and the Ways of Coping Questionnaire [47,48] have been used to measure different coping strategies in heterogeneous stressful situations and contexts and with many different population samples (e.g., patients, disaster survivors, health care workers).

In this article we will focus our attention on the COPE scale, [11], and, specifically, on its short version, i.e., on the Brief COPE scale [12].

### The Brief Cope scale

The original COPE scale is a multidimensional coping inventory developed to assess different ways in which people respond to stress [11]. Specifically, it aims to assess people’s active coping efforts, as well as “coping responses that may potentially impede or interfere with active coping” [11] (p. 280), and that may have dysfunctional quality. Its first version [11] is composed of 53 items: 13 scales, each with 4 items, plus 1 scale—substance use—with only 1 item. Its revised and complete form, which is accessible at the following link http://local.psy.miami.edu/faculty/ccarver/availbale-self-report-instruments/cope/ (last accessed 22 April 2022) [11], consists of 60 items, grouped in 15 faced-scales with 4 items for scale [12], which have been used to measure both dispositional and situational coping tendencies.

Its short version, the Brief-COPE (Coping Orientation to Problems Experienced) scale, is a self-report questionnaire consisting of 28 items, grouped in 14 faced-scales (each of them composed of 2 items), which represent 14 different coping reactions [12,13].

Like the long version, it was designed to measure different ways of coping with stressful events. The 28 items are measured with scores ranging from 1 (I haven’t been doing this at all) to 4 (I’ve been doing this a lot). Higher subscale scores indicate greater use of that coping strategy.

The Brief COPE can be used, like the long version, in three different formats: one dispositional and two time-limited (or situational). In the first one, that is the *dispositional* format, the respondents have to report the extent to which they *do* each of the things listed, when they are stressed. For this format, the verb form used for the items is the present tense (in the original English version). In the second one, that is one of the two *time-limited versions*, the respondents have to report the extent to which they *did* each of the things listed in a particular period in the past. In this case the verb form used for the item is the past tense. In the third format, that is the other *time-limited version*, the respondents have to indicate the extent to which they *have been doing* each of the things listed during a period up to the present. In this case, the verb form used for the item is the progressive or the present perfect [12] (pp. 95–98).

See S1 Table for the scale items in the present perfect form (as presented in the Brief COPE web page at link http://local.psy.miami.edu/faculty/ccarver/availbale-self-report-instruments/brief-cope/, last accessed 08/04/2022).

#### Faced scales (with no reversals of coding)

As said above, the 28 items are grouped in 14 faced-scales, each of which is composed of 2 items: Self-distraction (items 1 and 19), Active coping (2 and 7), Denial (3 and 8), Substance use (4 and 11), Use of emotional support (5 and 15), Use of instrumental support (10 and 23), Behavioral disengagement (6 and 16), Venting (9 and 21), Positive reframing (12 and 17), Planning (14 and 25), Humor (18 and 28), Acceptance (20 and 24), Religion (22 and 27), and Self-blame (13 and 26). See S1 Table among the Supporting Information for the specific content of each item.

In order to assess the internal structure of the Brief COPE and to permit the “correlations among factors”, Carver [12] conducted an exploratory factor analysis on the item set by using an oblique rotation, that yielded nine factors (Substance use; Religion; Humor; Behavioral Disengagement; Emotional support and Instrumental support; Active coping, Planning, Positive reframing (+ one item of acceptance); Venting and Self-distraction; Denial and Self-blame; Acceptance), revealing a remarkably similar structure to that identified for the full inventory [11]. Carver [12] validated this version of the scale on a relatively small sample of people, participating in a study on reactions to a cataclysmic event such as the natural disaster of Hurricane Andrew, which struck the Bahamas, Florida and Louisiana in 1992.

Although Carver prefers to focus on each scale separately, without identifying dominant coping styles and an overall coping index, he suggests, to the researchers who want to do that, to create second-order factors from among the scales [11]—to be used as predictors—as well as to use their own data, since different samples could show different patterns of relations.

Thus, Eisenberg et al. 2012 [53], for example (by carrying out an exploratory factor analysis with varimax rotation), identified in a sample of heart failure patients two major factors underlying the scale, i.e., two overarching coping styles: *approach coping* (active coping, planning, positive reframing, acceptance, seeking emotional support, seeking instrumental support) and *avoidant coping* (self-distraction, denial, venting, substance use, behavioral disengagement, self-blame). *Humor and religion* were excluded from these styles, since, according to the authors [53], have both adaptive and problematic components. Mache 2012 [54], instead, distinguished in a sample of hospital doctors three composite subscales: *problem-focused* (active coping, seeking instrumental support, planning), *emotion-focused* (acceptance, seeking emotional support, humor, positive reframing, religion), and *dysfunctional* (behavioral disengagement, denial, self-blame, self-distraction, substance use, venting).

Many validations of the Brief COPE scale have been carried out in numerous languages, among which Spanish [55]; French [56]; German [57]; Greek [58]; Korean [59].

Even though, the Brief COPE has been translated also into Italian [60] and used in different contexts and with different population samples (patients [61–63]; general population [6]; healthcare workers [64,65], also during the Covid-19 pandemic (healthcare workers [25,26,66,67]; patients [68], general population [69–71]), as far as we know, no validated version of the Italian situational Brief Cope inventory has been carried out. Indeed, although Monzani et al. 2015 [6], for example, investigated the dimensionality of an Italian situational version of the Brief COPE, they carried out their research on a general population sample, with the main aim to compare five different factor-models identified in previous studies, thus resorting solely to CFA. Therefore, as an Italian validation of the scale in its situational format (i.e., a version in which respondents have to indicate how often they have done each of the things listed by the items during a period ranging from the recent past to the current time when they are answering the questionnaire) is lacking in the literature, the present study aims to fill this gap, by investigating its dimensionality, i.e., by trying to identify its factor structure through exploratory and confirmatory factor analysis (EFA and CFA), on a large sample of Italian healthcare workers during the handling of a highly stressful situation such as a pandemic.

Out of the numerous scales for coping strategies assessment currently available, we chose the BRIEF-COPE one primarily because, as mentioned by Kato [46], it is one of the most widely used coping scale; its reliability has been widely proven through validations in languages other than English [55–59]; it is easy to administer, being a very short self-report questionnaire; it has already been applied in healthcare contexts [64,65], and in facing of cataclysmic stressful events (e.g., [12,65]). We therefore believe that it is an appropriate tool to probe the specific coping strategies adopted in response to a highly stressful event, such as the COVID-19 pandemic.

## Materials and methods

### Data collection

The present study was conducted in accordance with the ethical principles of the Declaration of Helsinki (https://www.wma.net/policies-post/wma-declaration-of-helsinki-ethical-principles-for-medical-research-involving-human-subjects/, last accessed on 01 June 2022), the APA Ethics Code (https://www.apa.org/ethics/code, last accessed on 01 June 2022) and the European and Italian Privacy Law (i.e., EU Reg. 679/2016, GDPR and Legislative Decree n. 196/2003, Code regarding the protection of personal data). It has been approved by the PhD meeting curriculum in Psychology, Communication, and Social Sciences, (University of Macerata. Protocol code n. 19435, 3 August 2020).

The data were collected through an online survey, which was run via LimeSurvey software (version 3.22; LimeSurvey GmbH, 2012 [72]) on a LAMP (Linux, Apache, MySQL, PHP) web-server. The HTTPS protocol and secure sockets layer (SSL) were adopted.

At the beginning of May 2020, an email was sent by the authors to the heads of the professional orders and of the main associations of Italian HCWs (nurses and physicians), providing them with information on the aims of the research (i.e., knowing the experiences of health care workers in relation to the COVID-19 emergency) and asking them to share the link for completing the questionnaire among their members and associates. Data collection started on May 15 and ended on 30 July 2020.

#### Inclusion criteria

Physicians and nurses’ members of the Italian professional orders and of the main associations, working in Italian hospitals, nursing and retirement homes, clinics in the territory etc., during the first wave of COVID-19.

#### Exclusion criteria

Physicians and nurses not enrolled in Italian professional orders; not on duty during the first wave of COVID-19; other healthcare workers different from physicians and nurses (e.g., psychologists, socio-health workers, secretarial staff, etc.).

The questionnaire was only administered to a sample of doctors and nurses because, although the pandemic affected the general population, the level of stress faced by them was certainly higher due to the greater risk of contagion. The questionnaire was not administered to other socio-health workers and psychologists because for the former, as far as we know, there is still no professional order or register in Italy to which we could have sent the request for the dissemination of the link for filling in the questionnaire (as was done for doctors and nurses), and for the latter because the number of those working in Italian health contexts is unfortunately rather low.

The snowball sampling method was used.

The questionnaire began with some details concerning the aims of the research (i.e., collecting data on Italians HCWs’ experiences in relation to the COVID-19 emergency), the identity and contact details of the research team, the planned way in which the results would be disseminated, and references to European and Italian laws on privacy and personal data protection. Respondents were able to start filling out the questionnaire after voluntarily consenting to participate by signing an online informed consent.

After a few questions, aimed at collecting socio-demographic, occupational and health worker exposure information on COVID-19, the IPSS-10 scale (Italian Perceived Stress Scale) and the Brief COPE scale were administered.

The IPSS-10 scale is the Italian version [73] of the PSS (Perceived stress scale). Although its original version consists of 14 items [74], the most used is that consisting of 10 items [75,76]. PSS measures the degree to which situations in one’s life are evaluated as stressful [77] (p. 1), by asking about feelings and thoughts during the last month. Respondents are asked how often they felt a certain way on a five-point Likert scale: 0 = Never; 1 = Almost Never; 2 = Sometimes; 3 = Fairly Often; 4 = Very Often.

Regarding coping, as we wanted to measure strategies used to deal with a specific stressful event (the spread of COVID-19), we presented the Brief COPE items in its situational format. We adapted its English original version into the Italian language using a forward and backward translation process, to guarantee correspondence between Italian and English. All the items of the questionnaire were compulsory. Each item was rated on a four-point scale (1 = I don’t agree at all; 2 = I agree a little bit; 3 = I agree on average; 4 = I agree a lot). The items were presented in a randomized manner. See S1 Table among the Supporting Information for the Italian translation of the Brief COPE inventory.

The estimated average time for compiling the socio-demographic questions, IPSS-10, and Brief COPE scale was approximately 10 minutes.

The database on which the statistical analyses were carried out was previously cleared of improbable answers. Responses from subjects who abandoned the questionnaire before the end were eliminated.

Minimal data set was deposited, under creative commons attribution license, in IEEE data repository, available online at the following link https://dx.doi.org/10.21227/fyzv-w953.

### Statistical analysis

As for the Brief COPE, following Carver’s recommendation (cf. https://local.psy.miami.edu/people/faculty/ccarver/availbale-self-report-instruments/brief-cope/), EFA and CFA were applied to our own data in order to determine the composition of the higher-order factors, since “different samples exhibit different patterns of relations”. In other terms, we used both EFA and CFA, as ours is a special sample (Italian health workers). The English version of the Brief Cope Scale was not built on this type of sample. Therefore, we felt it was necessary to study the factorial structure of our data using an EFA. It was important to determine the number of factors by being guided not only by theory, but also by data, following what is recommended in the bibliography. Both EFA and CFA treat the data as ordinal. The EFA uses a polychoric correlation and the CFA the DWLS (Diagonally Weighted Least Squares) estimator which are typical for ordinal data.

To test whether the obtained model was gender invariant and generalizable, invariance analysis was subsequently applied. By means of a “confirmatory multi-group factory analysis” [78] the “measurement invariance” is useful to study whether respondents from different groups interpret the same measure in a conceptually similar way [79]. We only considered the group variable “gender” because, among the variables surveyed, it is the only one that we believe can have a strong influence on coping strategies [80,81] and it is an unbalanced variable (approximately 77% females against 23% males; cf. the following section).

Finally, intercorrelation between I-Brief Cope and IPSS-10 was calculated to assess criterion validity. Since, IPSS-10 scale (Perceived Stress Scale) detects perceived stress, and perceived stress is related to the coping strategies a subject put in place, the IPSS-10 score can be used as a good measure for studying the criterion validity of I-Brief Cope, considering that the criterion validity measures how well one measure predicts an outcome for another measure [82].

### Sample characteristics

A total of 682 participants fully compiled the questionnaire: 530 (77.71%) were women and 152 (22.29%) men. Their mean age was 45.39 (ranging from 21 to 81, SD = 12.04). Many of them were married (45.01%) and had children (57.92%). Most of them declared to be religious (78.01%; specifically, practitioners: 15.84%; non-practitioners: 23.75%; only occasional practitioners: 38.42%). 75.95% worked as a nurse, and more than 70% in hospitals and care services in the northern regions of Italy (the area most affected by the first wave of the virus spread).

## Results

### Data analysis

All the analyses were carried out using R software, Version 4.2.0 (R Core Team, 2022 [83]).

McDonald’s omega for the Brief-COPE scale, calculated on our original data set, before EFA and CFA were carried out, was 0.837.

Usually, the database is divided in two: on the first half the EFA is calculated, and on the second half the CFA. In small samples, it is easy to show that the results of EFA and CFA depend on the particularity of the previous random selection.

To overcome this problem and to obtain more stable measures from the sample of 682 subjects (treated as population), 1000 different random samples of 341 subjects each (half the number of subjects) were extracted, on which the tests for the number of factors and then the Exploratory Factor Analysis (EFA) were calculated. This procedure makes it possible to obtain values that are more stable and not dependent on the sample selected. The same was done for the Confirmatory Factor Analysis (CFA), but on another 1000 different samples of 341 subjects, all different from the 1000 samples used for the EFA. In total, we used 2000 different samples (1000 for EFA and 1000 for CFA). This is only a minimal fraction of the possible different samples of 341 subjects that can be extracted from a sample of 682 subjects (equal to 6.128*10^203).

In other words, the results reported below for both EFA and CFA are the average of 1000 EFA and 1000 CFA calculated on 2000 different extracted samples.

For each of the 1000 extractions for the EFA, we first checked that the data were suitable for factor analysis. On average the Bartlett’s test of sphericity (Chi-sq (378) = 4143.112; p < 0.001) and the Kaiser-Meyer-Olkin test (KMO = 0.79) suggested that the data were suitable.

At a later stage, three different methods were used for each of the 1000 extractions for the EFA (Parallel analysis [84]; Velicer’s minimum average partial MAP, original version of 1976 [85] and revised version [86]) with the aim of determining the most likely number of factors to be set in the 1000 EFAs. Since the extractions show an average factor numerosity of 5.67, five and six-factors EFA were carried out.

Being our distribution “almost” bimodal (5 and 6), there was no substantial difference here between the use of the mode and the use of the mean. We examined both solutions, i.e., we calculated both a 5-factor and a 6-factor EFA. We used the mean to get an easy index that would tell us whether we were more skewed towards 5 or 6.

As the five-factor solution led to the loss of several items, which weighed on more than one factor at the same time and/or did not reach the minimum saturation value of 0.4, we decided to proceed with the six-factor solution, the results of which are given below.

A rule of thumb [87] for figuring out cross-loading is that you check the difference between the highest loading and the second highest loading for an item. If the absolute difference is < = 0.2, it means the item suffers from cross-loading. Ours was a conservative approach. Since an item should have a rotated factor loading of at least |0.4| (this is another rule of thumb) to be included in a factor, it suffers from cross-loading when the second highest loading is at least 0.2.

#### Exploratory factor analysis with 6 factors

The results of six-factor EFA (“Promax” oblique rotation) is shown in the following Table 1.

**Table 1:** EFA results.

ID	ITEM	F1	F2	F3	F4	F5	F6
7	Ho agito per cercare di migliorare la situazione.	**0,796**	-0,148	-0,068	-0,151	0,001	0,04
14	Ho cercato di trovare una strategia su cosa fare.	**0,761**	0,023	-0,069	-0,052	-0,031	-0,037
2	Ho concentrato i miei sforzi nel fare qualcosa per la situazione in cui mi trovo.	**0,717**	0,029	0,019	-0,207	0,003	0,031
25	Ho pensato molto a quali passi intraprendere.	**0,693**	0,121	0,026	-0,088	-0,001	-0,01
20	Ho accettato la realtà del fatto che è successo.	**0,482**	-0,211	-0,07	0,16	0,005	0,01
24	Ho imparato a conviverci.	**0,435**	-0,075	-0,03	0,137	-0,022	-0,026
9	Ho detto delle cose per far uscire i miei sentimenti spiacevoli.	0,099	**0,517**	0,095	-0,035	-0,069	0,026
8	Mi sono rifiutato/a di credere che sia successo.	-0,074	**0,508**	0,057	-0,052	-0,002	-0,058
13	Mi sono criticato/a.	0,169	**0,492**	0,123	0,011	-0,03	-0,024
16	Ho rinunciato al tentativo di farcela.	-0,248	**0,482**	0,075	0,003	0,095	0,088
6	Ho rinunciato a cercare di affrontarlo.	-0,21	**0,458**	0,041	0,118	0,005	0,037
26	Mi sono incolpato/a per le cose che sono successe.	0,033	**0,448**	0,083	0,003	0,007	0,027
15	Ho ricevuto conforto e comprensione da qualcuno.	-0,061	0,096	**0,751**	-0,021	0,002	0,017
10	Ho ricevuto aiuto e consigli da altre persone.	-0,046	0,118	**0,72**	-0,015	-0,025	-0,021
5	Ho ricevuto supporto emotivo da altri.	-0,062	0,129	**0,679**	-0,016	0,005	0,012
18	Ho fatto delle battute su questo.	-0,114	-0,035	-0,027	**0,938**	-0,032	0,001
28	Ho preso in giro la situazione.	-0,168	0,032	-0,009	**0,918**	0,014	0,004
22	Ho cercato di trovare conforto nella mia religione o nelle mie credenze spirituali.	-0,038	-0,029	-0,017	0,003	**0,767**	0,188
27	Ho pregato o meditato.	0,048	0,05	0,015	-0,05	**0,655**	0,146
4	Ho fatto uso di alcol o altre droghe per sentirmi meglio.	-0,008	0,082	0,054	0,016	0,169	**0,704**
11	Ho fatto uso alcool o altre droghe per aiutarmi a superare la situazione.	0,057	0,096	-0,021	0,002	0,18	**0,67**

As can be seen from Table 1, some items are missing. Specifically, the items

- 17, 21, 23, which load on two factors, i.e., which have a value of saturation of at least 0.2 on at least two factors;
- 1, 3, 12, 19, which have a saturation level lower than 0.4.
  - In other terms, the items eliminated are
- the items of the *self-distraction* scale: *1*. *I’ve been turning to work or other activities to take my mind off things; 19*. *I’ve been doing something to think about it less*, *such as going to movies*, *watching TV*, *reading*, *daydreaming*, *sleeping*, *or shopping;*
- the items of the *positive reframing* scale: *12*. *I’ve been trying to see it in a different light*, *to make it seem more positive; 17*. *I’ve been looking for something good in what is happening*
- one item of the *denial scal*e: *3*. *I’ve been saying to myself “this isn’t real”;*
- one item of the *venting scale*: *21*. *I’ve been expressing my negative feelings;*
- one item of the *instrumental support* scale: *23*. *I’ve been trying to get advice or help from other people about what to do*.

Eliminating these items results in “one-size-fits-all” (i.e., unambiguous) items, which load well on only one factor.

#### Confirmatory factor analysis

To test the factor structure, identified by EFA analysis, CFA (out of other 1000 different samples extracted, each of 341 subjects) has been conducted using the Diagonally Weighted Least Squares (DWLS) estimator.

We tested the adequacy of the confirmatory solutions by evaluating the following fit indices: the Root-Mean-Square Error of Approximation (RMSEA), the Comparative Fit Index (CFI), the Tucker-Lewis Index (TLI), the Standardized Root Mean Residual (SRMR), the Goodness of Fit Index (GFI), and the Adjusted Goodness of Fit Index (AGFI). The threshold values used to assess goodness-of-fit were: 0.08 for RMSEA and SRMR, 0.95 for TLI and GFI, 0.90 for CFI and AGFI. As per the literature [88–91], we considered only those models that showed fit indices below threshold values for RMSEA and SRMR and above threshold values for CFI, TLI, GFI, and AGFI to be good.

All the fit indices considered were good, averagely: CFI = 0.975; TLI = 0.971; RMSEA = 0.078; SRMR = 0.080; GFI = 0.980; AGFI = 0.968.

There are limitations with using the chi-square statistic as a model fit index. First, it is sensitive to sample size with larger sample sizes decreasing the p-value where there may only be a trivial misfit [92]. Overemphasis on model chi-square may lead to a preference for smaller samples in which the null hypothesis is not rejected. This is more likely to accept poor models and may yield inaccurate or imprecise parameter estimations [93]. Schermelleh-Engel et al. [94] suggest placing less emphasis on the chi-squared statistic. Joreskog and Sorbom [95] point out that chi-square should not be used as a formal statistical test. They recommend dividing the value of chi-sq by degrees of freedom (df). Adequate values of chi-sq/df are between 0 to 3. In our case, the average value of this ratio was 2.88.

**Fig 1:**
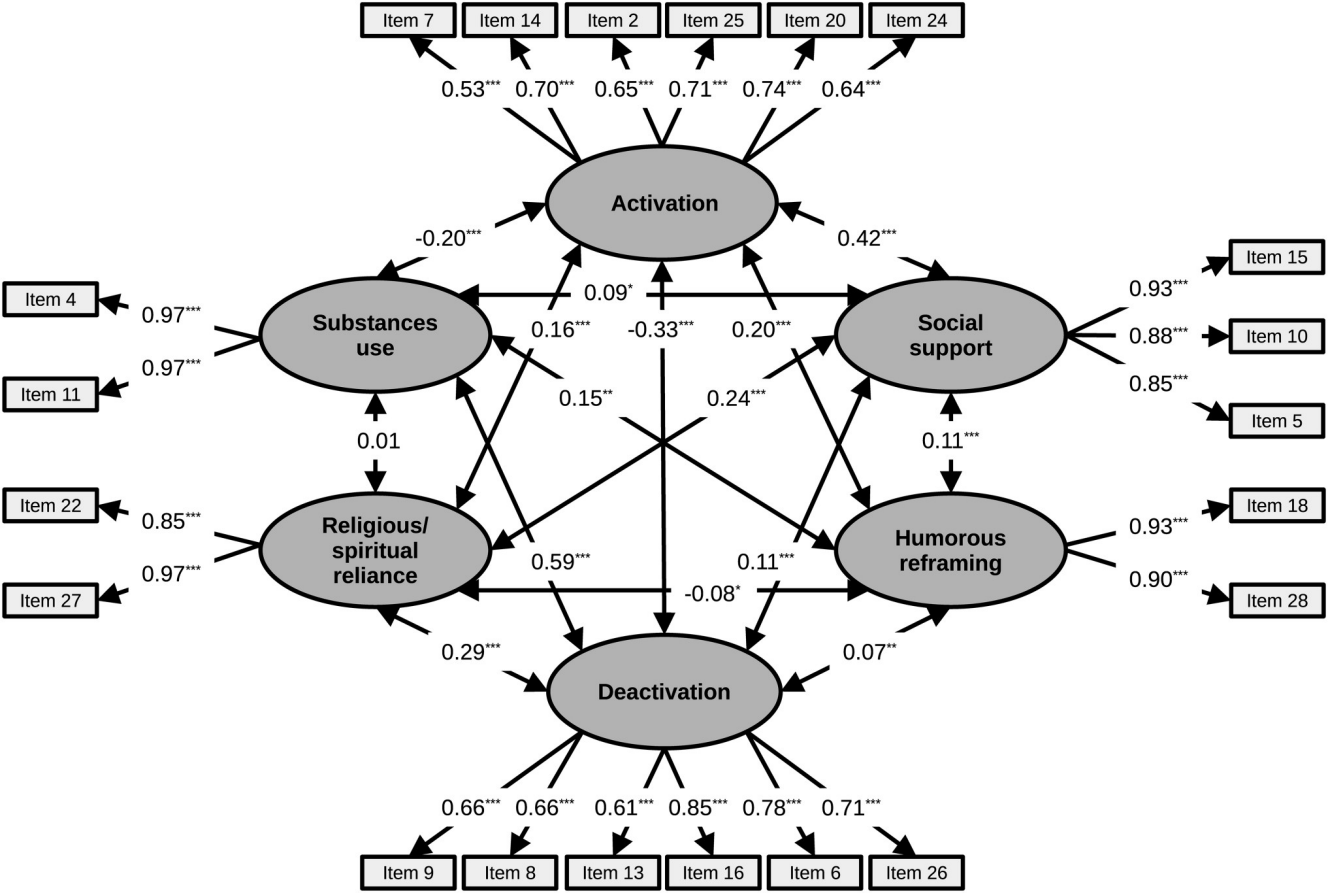
Factorial model of the I-Brief Cope scale. The digits represent standardized factor loadings. *p < 0.05; **p < 0.01; and ***p < 0.001. A two-way arrow between two variables indicates correlation.

Using the calculated RMSEA index as the effect-size and with an alpha of 0.05, the post-hoc power analysis says that a sample of 341 subjects is associated on average with a power of 99%. The following Fig 1 represents the path diagram for the CFA with standardized factor loadings.

Factor 1. Acceptance (20–24); Planning (14–25); Active coping (2–7) load on a single factor (F1). Taken together they form a relevant chain of what we can call the *subject’s activation*. In other terms, the subject accepts the situation, plans the actions, and then acts accordingly. Regarding the internal consistency of this factor, McDonald’s omega was 0.805.

Factor 2. Behavioral Disengagement (6–16); Self-blame (13–26); 1 of the 2 Denial items (8); 1 of the 2 Venting items (9) load on a single factor. In contrast to the previous factor, the items that load on F2 have to do with the *subject’s deactivation*: she or he manifests unpleasant emotions (item 9) and disbelief (item 8), blames themselves (items 13 and 26), refrains from action (items 6 and 16). Regarding the internal consistency of this factor, McDonald’s omega was 0.781.

Factor 3. Emotional support (5–15) and 1 of the two Instrumental support items (10) load on F3. F3 holds together items that have to do neither properly with an activation (as for the items in F1) nor with a deactivation (as for the items in F2) of the subject. The subject, in fact, obtains (because there is no active seeking-which is proper, on the other hand, to item 23, which does not load exclusively on this factor and which, therefore, has been excluded) from others emotional support, comfort and understanding, advice. In this sense, there is *social support*, i.e., *external support* for coping with the situation (the problem) and the related emotions. Regarding the internal consistency of this factor, McDonald’s omega was 0.882.

Three faced-scales (Humor, Religion, Substance use) form separate factors: F4, F5, F6.

Factor 4. The use of humor (items 18–28) in a condition such as a pandemic can be considered as a form of *cognitive reframing*. Regarding the internal consistency of this factor, McDonald’s omega was 0.858.

Factor 5. In *religious/spiritual reliance* (items 22–27), there is no activation on the part of the subject in coping with the problem, but an attempt to mitigate the emotional state resulting from the problem, seeking religious comfort, and relying on spiritual beliefs, perhaps also in the hope that the stressful situation (a cataclysmic event, with the consequent pain and deaths) will be resolved with divine help. Regarding the internal consistency of this factor, McDonald’s omega was 0.877.

Factor 6. Substance use (4–11) coincides with an implicit admission of inability to actively cope with a situation and its emotional consequences, except through the rescue of external substances (*addiction*). Unlike emotional or instrumental support (social support, F3) which comes from others (without an active search by the subject), substance use takes the form of *object-based support* (properly an addiction), actively sought, and assumed by the subject. Regarding the internal consistency of this factor, McDonald’s omega was 0.904.

In other terms, EFA and CFA revealed a second-order structure consisting of 6 factors, ranging from maximum activation (F1) to maximum deactivation (F2), via receiving social support from others (F3), humorous reframing (F4), religious/spiritual reliance (F5), and substance use (F6).

#### Invariance analysis

With the intention of testing whether the obtained model was invariant and generalizable with respect to the gender variable (i.e., to assess whether the mean values of males and females could be considered comparable), the models of three confirmatory multi-group factorial analyses, performed on the total of 682 subjects in the original sample, were compared.

“Measurement invariance” is useful to study whether respondents from different groups interpret the same measure in a conceptually similar way [79]. It is customary to calculate invariance on the sample on which the CFA was calculated, and often this sample corresponds to the entire sample. However, as our sample was unbalanced (77% were women), calculating invariance on 341 subjects would have meant reducing the number of men too much. We therefore calculated it on the entire sample of 682 subjects.

The three models were:

- *Configural model*: in which the same latent constructs were examined without imposing equality constraints between males and females;
- *Metric model*: in which the constraint of equality of factorial loadings between males and females was imposed;
- *Scalar model*: in which the constraint of equality of factorial loadings and intercepts between males and females was imposed.

To compare the three models, we considered the differences of the three indices, CFI, SRMR and RMSEA among the three multi-group models (configural vs metric and metric vs scalar). Invariance is verified if there is a difference of CFI less than or equal to 0.010, a difference of RMSEA less than or equal to 0.015, and a difference of SRMR less than or equal to 0.030 in the configural vs metric comparison and less than or equal to 0.010 in the metric vs scalar comparison [96]. The values in Table 2 testify to the presence of invariance by gender.

**Table 2:** Results of invariance analyses across gender (males, females).

Groups	Invariance model	RMSEA	CFI	TLI	SRMR	Invariance	ΔRMSEA	ΔCFI	ΔTLI	ΔSRMR
Gender	Configural	0.078	0.975	0.971	0.080		-	-	-	-
	Metric	0.078	0.973	0.971	0.082	Metric	0.000	-0.001	0.000	0.002
	Scalar	0.072	0.975	0.975	0.080	Scalar	-0.006	0.001	0.004	-0.001

#### Criterion validity

To assess the criterion validity, we calculated the intercorrelations between the I-Brief Cope and the IPSS (Italian Perceived Stress Scale [73]).

McDonald’s omega was 0.811.

In Table 3, the data show significant correlations between our coping dimensions and perceived stress. In particular, as perceived stress increases, the use of coping strategies increases.

**Table 3:** Intercorrelations (Pearson’s r) between the six dimensions of the I-Brief COPE and the IPSS-10. For the six dimensions of the I-Brief COPE, intercorrelations between scores and latent factors are reported.

		Perceived Stress	F1-Subject’s activation	F2-Subject’s deactivation	F3-External support	F4-Cognitive reframing	F5-Religious spiritual reliance
F1-Subject’s activation	Score	0.173***					
F2-Subject’s deactivation	Score	0.464***	-0.141***				
	Latent factor		-0.275**				
F3-External support	Score	0.229***	0.322***	0.151***			
	Latent factor		0.397**	0.170**			
F4-Cognitive reframing	Score	0.112**	0.199***	0.065	0.147***		
	Latent factor		0.249**	0.068	0.177**		
F5-Religious spiritual reliance	Score	0.208***	0.168***	0.203***	0.195***	-0.012	
	Latent factor		0.200**	0.248**	0.242**	-0.020	
F6-External substances (addiction)	Score	0.215***	-0.096*	0.436***	0.074	0.130***	0.031
	Latent factor		-0.115*	0.657**	0.79	0.186**	0.034

Note.

* p < .05,

** p < .01,

*** p < .001.

Considering that the “criterion validity” expresses the degree of correspondence between a measure and an external variable other than the original construct, the instrument generated criterion validity because the data showed consistently significant correlations between the I-Brief coping dimensions and perceived stress. When perceived stress increases, coping strategies increase.

Perceived stress (the IPSS-10 score) is always significantly related to the 6 dimensions derived. Considering the types of items, we can reasonably say that the 6 dimensions refer to coping strategies as they vary in direct proportion to stress.

## Discussion

In the present study the Italian situational Brief COPE inventory (I-Brief COPE) is presented. Its validation has been conducted on a sample of Italian healthcare workers employed during the first wave of the COVID-19 pandemic.

The EFA and CFA analyses were used and led to the identification of a specific structure made up of 6 second-order factors, on which 21 items out of the 28 of the original version loaded adequately.

Both the 6 identified dimensions (i.e., the second order factors), and the items of the scales that contribute to defining them, are not overlapping (if not partially) with what emerged either from the Brief-COPE original version [12]–according to which the 28 items loaded on 9 factors (substance use; religion; humor; behavioral disengagement; emotional and instrumental support; active coping, planning and positive reframing and 1 item of acceptance; venting and self-distraction; denial and self-blame; acceptance (1 item))–or from the validations carried out on languages other than English with other population samples [55–59]. The Spanish version of the BRIEF-COPE [55], applied to a sample of university students, led to the identification of 24 items loaded on 12 factors; the French version [56], administered to a sample of university students, led to the identification of functional and non-functional strategies and to the identification of gender-related differences according to which men would resort more than women to humour and substances use in coping with stress; the German version [57], administered to a sample of cataract surgery patients, led to the identification of 11 subscale summarized into 4 main factors; the Greek version [58], administered to an adult sample of the general population, led instead to the identification of 8 factors (4 larger and 4 smaller); the Korean one [59], administered to a sample of university students, led to the identification of 5 factors (problem focus coping; venting; avoidance; denial; humor).

The 6 factors that emerged from the present study bring together those strategies that seem to be the most readily understandable and relevant for the situation, not those most frequently used by our sample. In other terms, their identification does not mean that our sample specifically resorted to them (or that they did so more or less often); it simply means that the strategies falling in each of the factors identified can work in a particularly stressful situation such as a pandemic.

The six factors that emerged from this study are labelled:

***Subject’s activation*** (Acceptance; Planning; Active coping). This factor loads the items referring to coping strategies in which subjects actively intervene by accepting the situation, planning the actions, and acting. This factor is particularly pertinent for our sample of HCWs, who had to deal with a situation that was uncertain (because it was new) and poorly controllable and, for that reason, stressful both emotionally and jobwise.
***Subject’s deactivation*** (1 of the 2 Venting items; 1 of the 2 Denial items; Self-blame; Behavioral Disengagement). This factor loads the items referring to coping strategies in which subjects display unpleasant emotions and disbelief, blame themselves, and refrain from action.
***Social support*** (Emotional support; 1 of the 2 Instrumental support items). This factor loads the items referring to ways of coping with stress through others support. In other terms, the subjects merely recognize the external support as being functional in coping with the stressful event or the resulting emotions.

Consistently with Carver [12], three prior faced scales (humor, religion and substance use) form 3 distinct factors, we labelled:

***Humorous reframing*** (Humor). Subjects resort to humor in fronting the stress. This factor loads the items referring to humor, considered as a coping strategy due to its cognitive mechanism based on changing perspective on a demanding situation, namely its appraisal and reappraisal (e.g., [97,98]). The cognitive switch may impact positively on emotion regulation and positive emotions [99].
***Religious/spiritual reliance*** (Religion). This factor loads the items referring to religion or spiritual beliefs.
**Substance use (**Substance use). This factor loads the items referring to substance use, specifically, drugs and alcohol, but it would have been interesting to test whether other addictions (food, for example) fall properly under this factor, also in the light of data revealed by numerous studies concerning behavioral changes during repeated lockdowns (e.g., [100]).

In other terms, EFA and CFA revealed a second-order structure consisting of 6 dimensions, describing 6 different ways of coping with a particularly stressful event (such as the COVID-19 pandemic) by a sample of Italian HCWs. These strategies range from maximum activation (F1) to maximum deactivation (F2), via social support (F3), humorous reframing (F4), religious/spiritual reliance (F5), substance use (F6).

7 items (1, 3, 12, 17, 19, 21, 23) from the original version [12] do not load satisfactorily in any of the 6 factors (dimensions) identified, i.e., they do not seem adequate to measure HCWs’ coping strategies. Specifically, items 17, 21, 23 load on more than one factor (i.e., have a value of saturation of at least 0.2 on at least two factors), while items 1, 3, 12, 19 have a saturation level lower than 0.4.

It is not surprising that among the items that do not load properly there are those that Carver [12] labeled as *self-distraction* (1–19). Given the period under consideration (the first wave of COVID-19 in Italy) and the sample of subjects (HCWs), these strategies were probably the least suitable and perhaps the ones that could be least used (cf. item 19), also considering the work schedules and the hours spent in hospitals or in other healthcare facilities. Often, either to face the pandemic or for fear of infecting family members, especially during the first wave, many HCWs stayed in hospital for days. Item 1 (*I’ve been turning to work or other activities to take my mind off things*) was perhaps even less relevant than item 19, since the stressful event they had to cope with had to do properly with their work. In other terms, work could not be a distraction from the stressful problem. As for item 19, considering the context (the pandemic and the lockdown) and the impossibility of doing things that one generally does for distraction (going to the cinema or shopping), we could perhaps have rephrased item 19, adapting it to the circumstances. We could perhaps have indicated among the distraction activities: I watched TV, read, did online shopping, cooked, engaged in home hobbies etc. If the item had been reworded in this way, it would most probably have loaded better on one of the identified factors.

Similarly, the exclusion of items concerning the *positive reframing scale* (12–17) (Carver [12]) is not surprising and fits completely with the fact that it seems difficult to see in a different light (item 12) or to look for something good (item 17) in a serious situation, with a low level of controllability, as uncertain as that of the COVID-19 pandemic for HCWs, especially in the first waves of its spread. Also in this case, perhaps a rephrasing of the items to make explicit (using examples) what could be meant by “seeing in a different light” or “looking for something good” (e.g., “I focused on the positively resolved cases; the discharged patients; the teamwork, etc.”) would perhaps have allowed the items to load better on a factor.

Analogously, also the absence of item 3 of the *denial scale* seems to be reasonable, if we consider the sample. HCWs indeed had to deal with the concreteness of the virus, of its contagions, and the resulting deaths. The level of reality of this event was so intense as to make its denial very difficult. The other item of the same scale (item 8 *I’ve been refusing to believe that it has happened*)—which loaded properly on one of the factors (F2)- refers more to an attitude of disbelief. In other words, it could be understood as an attitude not of denial of the event (and/or its reality, as for item 3), but rather an attitude of surprise and astonishment towards its occurrence (contrary to expectations, probabilities). As if to say: “I never expected it to happen; it happened; I find it hard to believe it happened”.

Item 21 of the *venting scale* (*I’ve been expressing my negative feelings*) also does not load adequately in any factor. This absence is difficult to explain in consideration of the fact that item 9 of the same scale loads properly in one of the factors (F2). Perhaps, the absence could be due to the difference between *negative* (item 21) and *unpleasant* (item 9) feelings. While the manifestation of a negative emotion (often identified with anger, rage, etc.) is considered socially less appropriate and perhaps even less suitable as a coping response for an event such as a pandemic, an unpleasant emotion (often identified with sadness, a sense of loneliness) is considered a response more in line with the situation.

The last absence is that of item 23 (*I’ve been trying to get advice or help from other people about what to do*), which is one of the two instrumental support items. It was excluded because it weighed on two factors. Unlike items 5, 10, 15, which refer to having received emotional support, understanding, advice, item 23 differs because it implies a request (*I’ve been trying to get*…), which does not appear in the structure of the other three items. In other words, unlike the others, this item, in its very wording, describes an active support-seeking strategy, which in the case of the other items did not occur, as support was only received. Furthermore, in this item, unlike the other three, explicit reference is made to advice and help being sought on ‘what to do’, but the moment of great uncertainty made it difficult to seek guidance from others, considering that even authoritative voices in science were hesitating and sometimes disagreeing. Perhaps if the item had been rephrased as “I sought support by comparing my opinions with colleagues” it would have allowed the item to load better on the factor.

The latent dimensions we identified (and of the items loading on them) seem to be specific for the certain population sample we considered, as well as to the particular stressful event. However, we do not think that this means that the I-Brief Cope scale cannot be used with samples of Italian subjects other than health workers or in stressful situations other than those related to a pandemic event. This needs to be verified experimentally and therefore further studies with different samples are necessary. This, of course, does not mean that future research cannot be conducted in a direction that, on the contrary, more emphasizes contextual differences and is aimed at creating a more refined tool for measuring coping strategies that considers (even in the (re)formulation of the items) as much the specific stressful event as the different categories of individuals called upon to cope with its different effects.

The main limits of this research are procedural, and concern (1) having used a convenience sample (thus not representative of the Italian HCWs population), making the results non-generalizable and (2) having administered the questionnaire online. This may have allowed the same subject to fill out the questionnaire several times or a non-HCW to obtain the link to complete it. However, we believe that such eventualities are unlikely as the link was sent by the orders and associations to members’ personal email addresses and/or advertised on institutional websites.

Future research could move in the direction of creating more refined tools for measuring coping strategies in times of crisis, which would take into account (even in items formulation) both the specific critical or cataclysmic event and the different categories of individuals called upon to cope with the different stressful effects of the event under consideration.

## Conclusion

The Brief COPE has been administered on different population samples and translated into several languages. Nonetheless, the Italian validation of its situational format was missing, and the present study aimed to fill this gap. To this end, the original English version of the Brief-COPE scale was translated into Italian and administered to a sample of 682 Italian healthcare workers during the first wave of COVID-19.

The Exploratory and Confirmatory Factor Analysis (EFA and CFA) were performed on the collected data and led to a reduction in the number of items (21 items) compared to Carver’s [12] original version (28 items) and to the identification of a second level structure consisting of 6 main factors: Subject’s activation; Subject’s deactivation; Social support; Humorous reframing; Religious/spiritual reliance; Substance use (addiction). These strategies in stressful, highly unpredictable, and poorly controllable contexts are probably the most matched. Thus, for example, the complete exclusion of some prior faced scales, specifically those of self-distraction and positive reframing is not surprising and fits with the stressful situation considered and, to some extent, also with the sample to which the scale was administered. Repeated lockdowns made it impossible to perform some of the activities listed in item 19 (e.g., going to the cinema or shopping) or to consider work as an avoidance/distraction activity (item 1). As for the positive reframing, the pandemic and its consequences made it very complex.

To test whether the obtained model was invariant and generalizable, the invariance analysis was carried out, revealing invariance by gender. Finally, intercorrelation between I-Brief COPE and Italian Perceived Stress Scale (IPSS) was calculated, showing significant association between coping dimensions and perceived stress.

These results provide further evidence of the good psychometric properties of the Brief- COPE also when it is administered with a non-English-speaking samples and allow us to consider its Italian version (I-Brief COPE) as a valid tool for measuring coping strategies in facing stressful, unpredictable, and damaging events (such as the COVID-19 pandemic), among HCWs in stressful medical situations.

We think, nonetheless, that it could be a valuable instrument for assessing coping strategies among other population samples and in different stressful situations.

The Italian validation of the situational Brief COPE scale in a sample of Italian HCWs, engaged in the first wave of the COVID-19 pandemic, fills the gap identified in the literature.

Therefore, from a theoretical point of view, this study, on the one hand, confirms the soundness of an instrument that has already been validated in languages other than English, adding the Italian version to them (thus helping to add a small piece to the already existing literature); on the other hand, it confirms its author’s beliefs that different samples exhibit different patterns of relations.

From an applicative point of view, the results of this study can be useful for developing interventions to support the abilities of HCWs to cope with stressful public health-related events. HCWs who are better able to handle stressful situations—because they are trained in the use of positive coping strategies—help to improve the overall working climate as well as the effectiveness of their interventions on patients.

## Supporting information

S1 Table Original Brief-COPE items vs. I-Brief-COPE items. (DOCX)
