# Supplementary material for: Italian validation of the situational Brief Cope Scale (I-Brief Cope)
Source: PLoS One. 2022 Dec 1;17(12):e0278486. doi: 10.1371/journal.pone.0278486 (PMC9714734; doi:10.1371/journal.pone.0278486)
Supplement: S1 Table — (DOCX) [file pone.0278486.s001.docx]

**Supporting information**

**S1 Table. Original Brief-COPE items vs. I-Brief-COPE items.**

| **Items of the situational Brief-COPE (original version)*** | **Items of the situational I-Brief-COPE**** |
| --- | --- |
| 1. I’ve been turning to work or other activities to take my mind off things. | 1. Mi sono dedicato/a al lavoro o ad altre attività per distrarmi. |
| 2. I’ve been concentrating my efforts on doing something about the situation I’m in. | 2. Ho concentrato i miei sforzi nel fare qualcosa per la situazione in cui mi trovo. |
| 3. I’ve been saying to myself “this isn’t real”. | 3. Mi sono detto/a: “Questo non è reale”. |
| 4. I’ve been using alcohol or other drugs to make myself feel better. | 4. Ho fatto uso di alcol o altre droghe per sentirmi meglio. |
| 5. I’ve been getting emotional support from others. In q | 5. Ho ricevuto supporto emotivo da altri. |
| 6. I’ve been giving up trying to deal with it. | 6. Ho rinunciato a cercare di affrontarlo. |
| 7. I’ve been taking action to try to make the situation better. | 7. Ho agito per cercare di migliorare la situazione. |
| 8. I’ve been refusing to believe that it has happened. | 8. Mi sono rifiutato/a di credere che sia successo. |
| 9. I’ve been saying things to let my unpleasant feelings escape. | 9. Ho detto delle cose per far uscire i miei sentimenti spiacevoli. |
| 10. I’ve been getting help and advice from other people. | 10. Ho ricevuto aiuto e consigli da altre persone. |
| 11. I’ve been using alcohol or other drugs to help me get through it. | 11. Ho fatto uso di alcool o altre droghe per aiutarmi a superare la situazione. |
| 12. I’ve been trying to see it in a different light, to make it seem more positive. | 12. Ho cercato di vedere la situazione sotto una luce diversa, per farla sembrare più positiva. |
| 13. I’ve been criticizing myself. | 13. Mi sono criticato/a. |
| 14. I’ve been trying to come up with a strategy about what to do. | 14. Ho cercato di trovare una strategia su cosa fare. |
| 15. I’ve been getting comfort and understanding from someone. | 15. Ho ricevuto conforto e comprensione da qualcuno. |
| 16. I’ve been giving up the attempt to cope. | 16. Ho rinunciato al tentativo di farcela. |
| 17. I’ve been looking for something good in what is happening. | 17. Ho cercato qualcosa di buono in ciò che sta accadendo. |
| 18. I’ve been making jokes about it. | 18. Ho fatto delle battute su questo. |
| 19. I’ve been doing something to think about it less, such as going to movies watching TV, reading, daydreaming, sleeping, or shopping. , | 19. Ho fatto qualcosa per pensarci meno, come andare al cinema, guardare la TV, leggere, sognare ad occhi aperti, dormire o fare shopping. |
| 20. I’ve been accepting the reality of the fact that it has happened. | 20. Ho accettato la realtà del fatto che è successo. |
| 21. I’ve been expressing my negative feelings. | 21. Ho espresso i miei sentimenti negativi. |
| 22. I’ve been trying to find comfort in my religion or spiritual beliefs. | 22. Ho cercato di trovare conforto nella mia religione o nelle mie credenze spirituali. |
| 23. I’ve been trying to get advice or help from other people about what to do. | 23. Ho cercato di ricevere consigli o aiuto da altre persone su cosa fare. |
| 24. I’ve been learning to live with it. | 24. Ho imparato a conviverci. |
| 25. I've been thinking hard about what steps to take. | 25. Ho pensato molto a quali passi intraprendere. |
| 26. I’ve been blaming myself for things that happened. | 26. Mi sono incolpato/a per le cose che sono successe. |
| 27. I’ve been praying or meditating. | 27. Ho pregato o meditato. |
| 28. I‘ve been making fun of the situation. | 28. Ho preso in giro la situazione. |

* *Scores*  (1 = I haven't been doing this at all; 2 = I've been doing this a little bit; 3 = I've been doing this a medium amount; 4 = I've been doing this a lot).

*** Scores* (1 = I don’t agree at all; 2 = I agree a little bit; 3 = I agree on average; 4 = I agree a lot).
